# Bilateral Chylothorax and Chylous Ascites: A Rare Presentation of an Uncommon Disorder

**DOI:** 10.7759/cureus.14044

**Published:** 2021-03-22

**Authors:** Akhilesh Mahajan, Pratyaksha Sankhyan, Boonphiphop Boonpheng

**Affiliations:** 1 Pulmonary Critical Care, Lenox Hill Hospital, New York, USA; 2 Internal Medicine, East Tennessee State University, Johnson City, USA; 3 Pulmonary Critical Care, East Carolina University, Greenville, USA; 4 Nephrology, University of California Los Angeles, Los Angeles, USA

**Keywords:** chylothorax, chylous ascites, follicular lymphoma, peritoneal carcinomatosis

## Abstract

We describe the case of a 62-year-old female who presented with gradually progressing abdominal distension and dyspnea. Computed tomography (CT) chest and abdomen revealed large bilateral pleural effusions with large ascites, a mid-abdominal mass, and peritoneal carcinomatosis. Pleural and peritoneal tap revealed chylous fluid, and the biopsy findings from abdominal mass were consistent with follicular lymphoma. We then discuss a review of the literature and diagnoses for bilateral chylothorax and chylous ascites.

## Introduction

Chylothorax and chylous ascites are both rarely encountered and often difficult-to-manage clinical entities with only a few case reports and even fewer clinical studies. Both of these are usually associated with malignancies, however, chylothorax and chylous ascites as the presentation of malignancy are rare.

Follicular lymphoma is the most common type of indolent non-Hodgkin lymphoma. It is a very slow-growing lymphoma that generally affects the lymph nodes and may spread to the bone marrow or spleen. Patients commonly present with gradually enlarging painless peripheral lymphadenopathy and the diagnosis is usually made on a lymph node biopsy.

We describe an important presentation of this disease in a patient who had no peripheral lymphadenopathy but instead had both chylothorax and chylous ascites caused by the lymphoma, which kept growing insidiously until she presented with abdominal distension and dyspnea. Pleural and peritoneal fluid cytology and flow cytometry were negative for malignant cells and definitive diagnosis could only be obtained on fine-needle aspiration and core needle biopsy of her large mid-abdominal mass.

## Case presentation

A 62-year-old caucasian female with no significant past medical history presented to our hospital with the chief complaint of gradually progressing abdominal distension for one month and progressive dyspnea with a decreased appetite for five to seven days. She was released about two months ago from a penitentiary and had not been seen by a physician in several years. On review of systems, she denied any fever, chills, night sweats, or weight loss. 

On physical examination, she was hemodynamically stable with normal oxygen saturation on room air. She had dullness to percussion in the scapular line till the inferior angle of the scapula on both sides and decreased breath sounds at both lung bases. A grossly distended abdomen with shifting dullness was also noted. She didn’t have any palpable lymphadenopathy and the rest of her physical examination was normal.

Her laboratory data on admission were within normal limits with a white cell count (WCC) of 5.7 K/uL, hemoglobin of 13 g/dL, platelet count of 381 K/uL, and international normalized ratio (INR) of 1.0. Her electrolytes, liver function tests, and renal function tests were also within normal limits.

**Figure 1:**
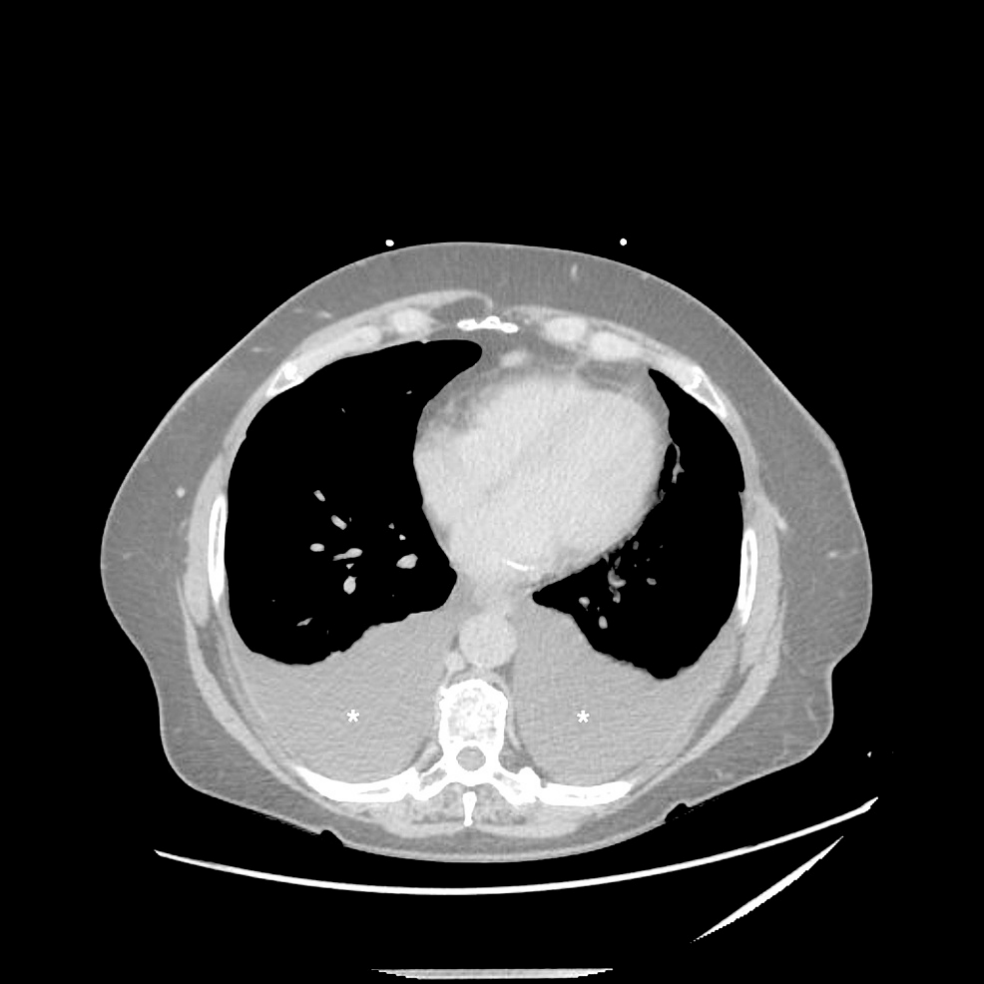
Computed tomography of chest (axial section) CT chest demonstrates large bilateral pleural effusions (white asterisks)

**Figure 2:**
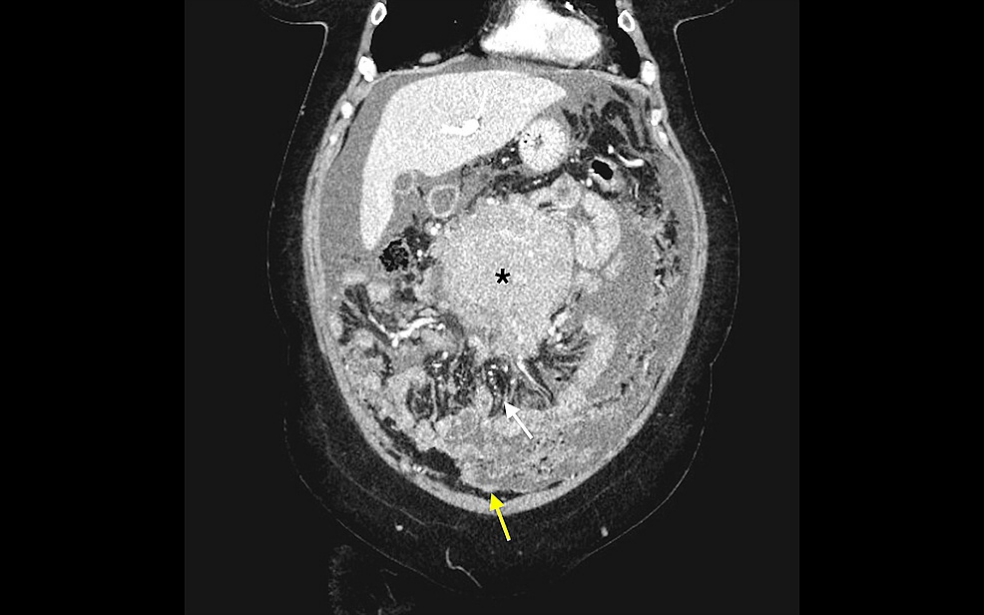
Computed tomography of the abdomen (coronal section) CT abdomen describes a large mid-abdominal mass (black asterisk), mesenteric thickening (yellow arrow), and peritoneal thickening with stellate appearance (white arrow) suggesting peritoneal carcinomatosis.

**Figure 3:**
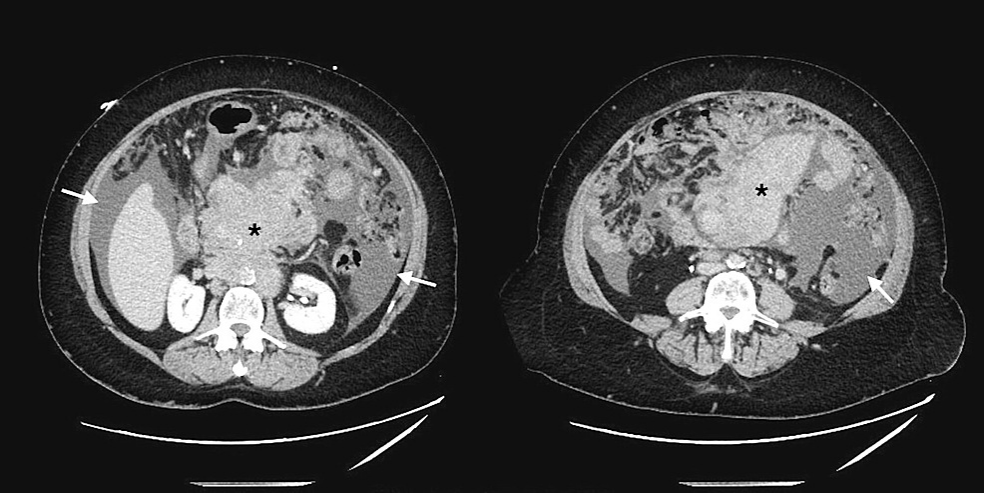
Computed tomography of the abdomen (axial section) CT abdomen demonstrates a large midabdominal mass (black asterisks) and large ascites (white arrows).

Computed tomography (CT) of the chest and abdomen with contrast revealed large bilateral pleural effusions with volume loss at the lung bases (Figure 1), large ascites with a mid-abdominal mesenteric mass, measuring 14 cm x 9 cm x 13 cm, engulfing the superior mesenteric artery (Figure 2) and possible peritoneal carcinomatosis (Figure 3). Her liver, spleen, and ovaries were noted to be normal. At this point, the differentials included lymphoma versus Kruckenberg tumor versus a number of gastrointestinal tract malignancies that could present as peritoneal carcinomatosis.

Under aseptic precautions, ultrasound (US)-guided paracentesis and left-sided thoracentesis were performed and 1100 mL and 500 mL of cloudy white fluid were drained, respectively, with significant improvement in the patient’s symptoms. The pleural and peritoneal fluid laboratory parameters have been presented in Table 1.

**Table 1:** Pleural and peritoneal fluid analysis lactate dehydrogenase (LDH); white cell count (WCC)

Lab parameters	Serum	Pleural fluid	Peritoneal fluid
Protein	6.2 g/dL	4.3 g/dL	4.3 g/dL
Albumin	3.3 g/dL	3.2 g/dL	3.1 g/dL
LDH	393 U/L	345 U/L	
Triglycerides		965 mg/dL	
Cholesterol		137 mg/dL	
Glucose	93 mg/dL		
WCC		4606 cells/uL	2261 cells/uL

On chemistry, pleural fluid was exudative in nature as per Light’s criteria with an effusion-to-serum protein ratio of more than 0.5 and an effusion-to-serum lactate dehydrogenase (LDH) ratio greater than 0.6. Peritoneal fluid was also exudative in nature with serum ascites albumin gradient (SAAG) less than 1.1 g/dL. On cytology, lymphocyte predominance was seen with no malignant cells. Gram stain, acid-fast stain, and cultures were also negative. Immunophenotypic analysis of pleural and peritoneal fluid by flow cytometry showed polyclonal lymphocyte population without clonal excess and no immunoglobulin light chain restriction, aberrant antigenic expression, or antigen loss. She also had a normal CD4: CD8 ratio of 3:1 and B lymphocytes with a normal Kappa: Lambda light chain ratio of 1:2.

Despite the presence of a large intraabdominal mass, peritoneal and pleural fluid flow cytometry remained inconclusive and hence CT-guided fine-needle aspiration (FNA) and core needle biopsy were done. While awaiting the biopsy results, she was switched to a high protein and low-fat diet with medium-chain triglyceride (MCT) supplementation. Since the patient was symptomatically better and no recurrent fluid collection over the following two days was seen, she was discharged home with a close outpatient follow-up.

FNA and biopsy results were available six days later and revealed that approximately 29% of lymphocytes contained a monoclonal B cell lymphoid population with intermediate to large cell size with lambda immunoglobulin light chain restriction. Results of flow cytometry and fluorescent in situ hybridization (FISH) study are provided in Table 2.

**Table 2:** Flow cytometry and FISH results B-cell lymphoma-6 (BCL-6); fluorescent in situ hybridization (FISH)

Markers present	Markers absent
CD45	CD5
CD19	CD23
CD20	
CD10	
CD38	
FMC7	
Translocation present	Translocation absent
Translocation 14;18 in 41% of the cells	BCL-6 and MYC gene

On the basis of clinical features and biopsy results, a diagnosis of stage IV follicular lymphoma was made, and she was scheduled to follow up with oncology as an outpatient for further management.

## Discussion

Epidemiology and etiology

**Figure 4:**
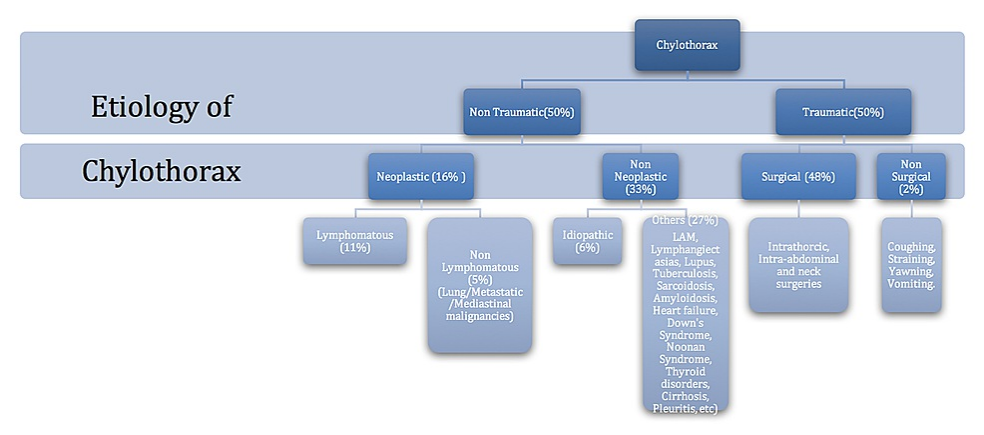
Etiology of chylothorax Source: [1]

Both chylothorax and chylous ascites are rare clinical entities defined by the presence of lymphatic fluid in the pleural and peritoneal cavities, respectively. A retrospective study done at Mayo Clinic, Rochester, in 2005 revealed a total of only 203 cases of chylothorax in the past 21 years with lymphomas being an important cause of nontraumatic chylothorax [1]. In a large collective study by Aalami et al. in 2000, the most common etiologies for chylous ascites included lymphomas and cirrhosis, which accounted for over two-thirds of their studied patients [2-3]. Figure 4, adapted from [1], lists important causes of chylothorax.

Pathogenesis

**Figure 5:**
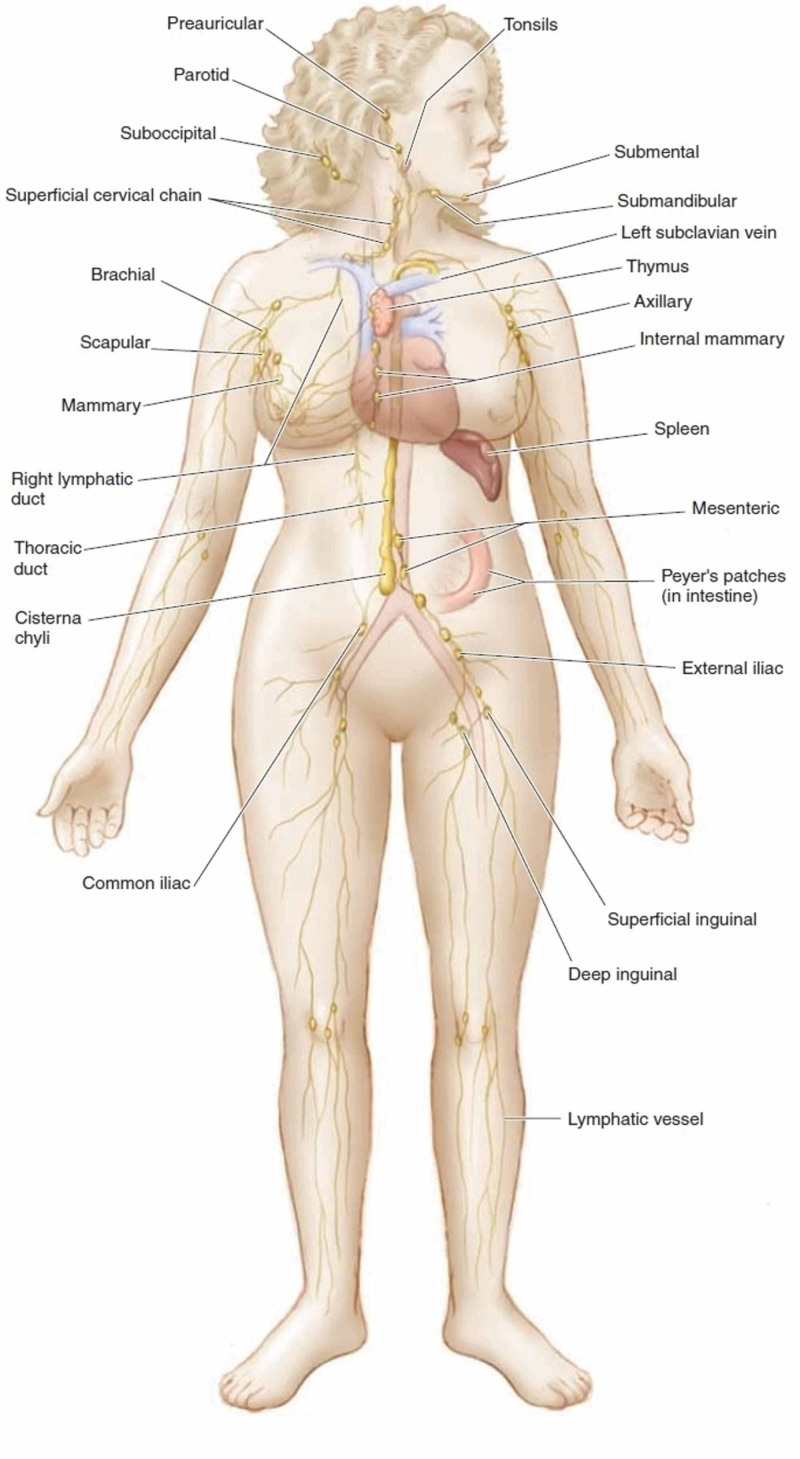
Normal lymphatic drainage Source: [5]

In a healthy adult, the thoracic duct transports 1.5 to 4 L of chyle daily, and its obstruction can cause leakage of chyle through lymphoperitoneal fistulae or leakage through dilated subserosal lymphatics into the peritoneal cavity [4]. The normal lymphatic system is depicted in Figure 5. In our patient, direct invasion of the cisterna chyli by the abdominal mass leading to chylous exudation into the peritoneum and further tracking up of this exudate across the diaphragm via diaphragmatic defects causing bilateral chylothorax seems to be the plausible mechanism.

Diagnosis

For the diagnosis of this condition, thoracentesis and paracentesis with fluid analysis remain the most important tools. Chyle has typically been described as a whitish exudate with a “milky,” cloudy appearance due to its high triglyceride content. However, Staats et al. in 1980 [6] and Maldonado et al. in 2009 [7] demonstrated that less than 50% of chylous pleural effusions (proved by lipoprotein analysis) had this characteristic appearance with the majority being serous and serosanguinous in appearance. This emphasizes the importance of obtaining triglyceride and cholesterol levels in all pleural effusions of undetermined causes regardless of appearance [6-7]. Triglyceride values greater than 110 mg/dL are highly suggestive of chylous effusion, whereas levels less than 50 mg/dL are unlikely to be chylous effusions. Equivocal cases between 50 and 110 mg/dL require lipoprotein analysis for the demonstration of chylomicrons [8]. Maldonado et al. also noted that a majority (86%) of these effusions were exudative due to the pleural fluid: serum protein ratio being more than 0.5. The occasional transudative chylous effusions were most commonly seen with ascites. In all the chylous effusions in this study, LDH levels were usually in the transudative range, which is in contrast to our patient with an LDH of 345 U/L, likely due to her large midabdominal follicular lymphoma. Most chylous effusions are lymphocyte-predominant due to the presence of T lymphocytes in the lymph while neutrophil predominance is mostly seen in post-surgical cases or in association with intraperitoneal infections [7]. Our patient’s pleural effusion on tapping had a characteristic whitish milky appearance with a triglyceride level of 965 mg/dL and lymphocytic predominance.

Management

The management strategies and prognosis for chylous ascites and pleural effusions largely depend on the underlying etiology, and a wide variation in resolution rates has been noted. Conservative management with a high-protein and low-fat diet, which is rich in MCTs, is recommended since MCTs are absorbed by the intestinal cells and transferred as free fatty acids (FFA) and glycerol directly to the liver via the portal vein with no involvement of the thoracic duct, thereby decreasing the volume of chyle. However, MCT supplementation is contraindicated in patients with advanced cirrhosis due to the high risk of narcosis and subsequent coma [9]. Repeated peritoneal and pleural taps, along with chest tube drainage, are often needed for symptomatic treatment.

In 2006, Roehr et al. reported a study describing the use of somatostatin and octreotide drips for the management of chylothorax in 35 children. This approach is often combined with fasting and total parenteral nutrition (TPN) as a second-line treatment in case of the failure of conservative management [10]. Maldonado et al., in a retrospective study of 74 patients, noted that conservative management was successful for 49% of patients with traumatic chylothorax but only 27% in nontraumatic chylothorax [11]. In such cases, surgical modalities, including thoracic duct ligation or embolization, remain the last line of defense and might actually be preferred in patients with especially large drainage of more than a liter in a day.

Our patient had very slowly enlarging chylous ascites and effusion, which were easily managed with therapeutic thoracentesis, paracentesis, and dietary modifications. She was referred to oncology for definitive treatment of her follicular lymphoma.

## Conclusions

Chylothorax and chylous ascites are rare clinical entities, and lymphoma is the most common nontraumatic etiology for both and should be ruled out in these patients. Gross inspection of the pleural and peritoneal fluid is the first clue in diagnosis but can often be misleading. Hence, chemical analysis of pleural fluid and ascites of unknown etiology should always include triglyceride levels. Lipoprotein analysis should be considered for triglycerides between the range of 50 to 110 mg/dL. A trial of conservative management with dietary changes should be given before proceeding to more invasive procedures, especially in nontraumatic cases with a slow rate of re-accumulation.
